# Exploring necrotizing autoimmune myopathies with a novel immunoassay for anti-3-hydroxy-3-methyl-glutaryl-CoA reductase autoantibodies

**DOI:** 10.1186/ar4468

**Published:** 2014-02-03

**Authors:** Laurent Drouot, Yves Allenbach, Fabienne Jouen, Jean-Luc Charuel, Jérémie Martinet, Alain Meyer, Olivier Hinschberger, Brigitte Bader-Meunier, Isabelle Kone-Paut, Emmanuelle Campana-Salort, Bruno Eymard, Anne Tournadre, Lucile Musset, Jean Sibilia, Isabelle Marie, Olivier Benveniste, Olivier Boyer

**Affiliations:** 1 Inserm, U905 & Normandie Univ, IRIB, 22 Bd Gambetta, F-76000 Rouen, France; 2 Pierre and Marie Curie University & Inserm, U974 & Assistance Publique - Hôpitaux de Paris, Pitié-Salpêtrière University Hospital, Department of Internal Medicine, Paris, France; 3 Rouen University Hospital, Department of Immunology, Rouen, France; 4 Assistance Publique - Hôpitaux de Paris, Pitié-Salpêtrière University Hospital, Laboratory of Immunochemistry & Autoimmunity, Paris, France; 5 Department of Physiology, Nouvel Hôpital civil, Strasbourg, France; 6 Department of Internal Medecine, Mulhouse Hospital, Mulhouse, France; 7 Inserm, U768, Paris, France; 8 Department of Immunology and Hematology, Assistance Publique - Hôpitaux de Paris, Necker University Hospital, Paris, France; 9 Assistance Publique - Hôpitaux de Paris, Bicêtre University Hospital, Department of Pediatrics, Le Kremlin-Bicêtre, France; 10 Assistance Publique - Hôpitaux de Marseille, La Timone University Hospital, Marseille, France; 11 Assistance Publique - Hôpitaux de Paris, Pitié-Salpêtrière University Hospital, Institute of Myology, Paris, France; 12 Department of Rheumatology, Clermont-Ferrand University Hospital, Clermont-Ferrand, France; 13 Rouen University Hospital, Department of Internal Medicine, Rouen, France

## Abstract

**Introduction:**

Necrotizing autoimmune myopathies (NAM) have recently been defined as a distinct group of severe acquired myopathies, characterized by prominent myofiber necrosis without significant muscle inflammation. Because of the lack of appropriate biomarkers, these diseases have been long misdiagnosed as atypical forms of myositis. NAM may be associated to autoantibodies directed against signal recognition particle (SRP) or 3-hydroxy-3-methyl-glutaryl-CoA reductase (HMGCR). The objective of this work was to quantify anti-HMGCR autoantibodies in patients with suspicion of NAM through the development of a new addressable laser bead immunoassay (ALBIA).

**Methods:**

Recombinant HMGCR C-domain was bound to fluorescent beads. After incubation with serum, autoantibodies were revealed using class- or subclass-specific anti-human immunoglobulin G (IgG) antibodies. Anti-HMGCR levels were assayed in 150 patients with suspicion of NAM, 142 controls with different inflammatory/autoimmune diseases and 100 healthy donors. Inhibition with free recombinant HMGCR and immunoprecipitation experiments confirmed test specificity. Reproducibility and repeatability were determined from sera with various levels of anti-HMGCR autoantibodies. A multiplex assay (ALBIA-NAM) was also developed to permit the simultaneous quantification of anti-HMGCR and anti-signal recognition particle autoantibodies.

**Results:**

No controls scored positive. Of 150 patients with suspicion of NAM, 24% were positive for anti-HMGCR autoantibodies with levels ranging from 24 to 2,656 AU/mL. Anti-HMGCR positivity could be associated to a cytoplasmic pattern in immunofluorescence assay on HEp-2 cells. Anti-HMGCR-positive patients had high creatine kinase (CK) levels (mean 6,630 IU/L) and only 40% of them had been exposed to statins. Multiplex ALBIA-NAM was equally as effective as monoplex anti-HMGCR and anti-SRP ALBIA.

**Conclusions:**

Both monoplex ALBIA-HMGCR and multiplex ALBIA-NAM reliably detect and quantify anti-HMGCR autoantibodies. A positive result allows ascribing patients with a necrotizing myopathy to an autoimmune form. Anti-HMGCR autoantibodies may be found in patients who have not taken statins.

## Introduction

Inflammatory myopathies are a heterogeneous group of acquired muscle disorders including polymyositis, dermatomyositis, inclusion body myositis and overlap myositis. Recently, necrotizing autoimmune myopathies (NAM) have been defined as a distinct group of severe acquired myopathies, characterized by pathological features of prominent myofiber necrosis without significant inflammation [1]. Because of the lack of appropriate biomarkers, these diseases have been long misdiagnosed as atypical forms of myositis with little if any inflammation [2-4]. With the emergence of reports describing clinical cases of necrotizing myopathy with microangiopathy and microvascular deposition of complement [5], they were progressively distinguished from myositis and finally classified as NAM by a collaborative study group in 2004 [6]. NAM may be associated with autoantibodies (aAbs) such as anti-signal recognition particle (SRP) autoantibodies. Anti-SRP aAbs are present in a minority (4 to 6%) of patients with acquired inflammatory and/or necrotizing myopathies [6-10] and are associated with severe clinical forms, particularly with heart involvement [11,12]. In 2007, Needham *et al.* reported eight patients who developed a myopathy during statin therapy [13]. Histological analysis of muscle biopsies revealed necrotic and regenerating myofibers. Later, aAbs against a 100 kDa protein were characterized and finally identified by Mammen *et al*. as directed against 3-hydroxy-3-methylglutaryl-coenzyme A reductase (HMGCR), a key enzyme of the cholesterol biosynthesis pathway, which is pharmacologically inhibited by statins [14,15].

The diagnosis of NAM may be difficult, notably in slowly progressive forms in young adults, since histopathological features mimic some forms of muscular dystrophies and some patients may remain without diagnosis for years. Hence, the detection of aAbs in patients with undetermined necrotizing myopathy is of utmost importance to indicate an autoimmune mechanism and therefore guide appropriate therapy. Currently, the detection of anti-SRP autoantibodies is based on indirect immunofluorescence on HEp-2 cells, followed by confirmation using a dot-blot test or a quantitative addressable laser bead immunoassay (ALBIA) [16,17]. Here, we developed a new ALBIA for the detection and titration of anti-HMGCR aAbs (ALBIA-HMGCR) as a biomarker of NAM. Also, in order to provide a unique test for the diagnosis of aAb-associated NAM, we further developed a multiplex assay (ALBIA-NAM) and determined whether it could discriminate patients with anti-HMGCR and anti-SRP aAbs.

## Material and methods

### Serum samples

In this medical research, where we used identifiable human sera and data, we obtained agreement from the French Ministry of Research (AC 2008-87) for the collection, analysis, storage and reuse. All samples were obtained for diagnostic purposes only with no extra sampling and, in this situation of retrospective study, patients’ consent was not required. The research protocol was approved by research ethics committee of Pitié-Salpêtrière University Hospital.

Serum or blood samples (n = 150 patients) that were sent to the Laboratory of Immunology of Rouen University Hospital for analysis of anti-HMGCR aAbs because of suspicion of NAM were analyzed. These samples were obtained between August 2012 and January 2013 from 24 French centers and were all negative for anti-Jo-1 and anti-SRP aAbs. All patients who were found positive for anti-HMGCR aAbs were negative for anti-PL-7 and anti-PL-12 aAbs. For these anti-HMGCR-positive patients, the following parameters were recorded from their medical reports: age, sex, past medical history, statin exposure, characteristics of myopathy including clinical manifestation and muscular deficit evaluation by muscle manual testing using the Medical Research Council scale on five points, and creatine kinase (CK) level at the date of the anti-HMGCR assay. Special emphasis was performed by the clinician to check statin exposure during follow-up medical consultations. For correlation experiments, we additionally analyzed 25 anti-SRP-positive sera that were described previously [16]. The term SRP in text and figures refers to the 54 kDa subunit of the multimeric SRP complex.

Control sera were collected from 100 healthy blood donors and from 142 patients with different inflammatory/autoimmune diseases, according to established classification criteria: American College of Rheumatology (ACR) revised criteria for systemic lupus erythematosus (SLE) [18] with anti-double-stranded DNA (dsDNA) aAbs (n = 19), American Rheumatism Association (ARA) criteria for rheumatoid arthritis (RA) [19] with anti-cyclic citrullinated peptide (CCP) antibodies and/or rheumatoid factor (n = 18), revised European criteria for primary Sjögren’s syndrome [20] with anti-SSA and/or anti-SSB aAbs (n = 15), Bohan and Peter criteria [21] for dermatomyositis (DM) (n = 8), Troyanov criteria for overlap myositis [10] with anti-tRNA-synthetase Ab (anti-Jo-1, n = 27; anti-PL-7, n = 1; anti-PL-12, n = 2) and Griggs criteria for inclusion body myositis (IBM) [22] (n = 30). Another control consisted in sera from patients with polyclonal hypergammaglobulinemia (serum immunoglobulin (Ig)G: 23.1 ± 6.9 g/L, mean ± standard deviation (SD)) (n = 24). All serum samples were stored at -80°C until use.

### Western blot analysis of recombinant C-terminal domain of the HMGCR protein

The catalytic C-domain (amino acids 441 to 880) of human HMGCR protein fused to a glutathione S-transferase (GST) tag was obtained from Sigma-Aldrich (St Louis, MO, USA). Purity of this recombinant HMGCR protein was first determined by 4 to 10% gradient sodium dodecyl sulfate polyacrylamide gel electrophoresis (SDS-PAGE) under nonreducing conditions, followed by Coomassie Blue staining. Western blot analysis was further performed by transfer of proteins separated by nonreducing SDS-PAGE to a nitrocellulose membrane followed by incubation with rabbit anti-HMGCR Abs (Abcam, Cambridge, MA, USA) or a human anti-HMGCR-positive serum and revelation with corresponding secondary antibody coupled to Alexa Fluor 680 (Invitrogen, Cergy Pontoise, France).

### Immunoprecipitation of human recombinant HMGCR with patients’ sera

Immunoprecipitation was performed by adding 20 μl of human serum sample or 1 μg of positive control rabbit anti-HMGCR Ab to 10 μg of recombinant HMGCR C-domain and bringing the volume to 0.5 ml of IP buffer (1% Nonidet P40, 20 mM Tris pH 7.4, 150 mM NaCl, 1 mM EDTA) and rotating the mix for 2 h at 4°C. Protein G agarose beads (Pierce, Rockford, IL, USA) were added for an additional hour. Then, immunoprecipitates were boiled in the presence of Laemmli buffer and subsequently electrophoresed on 4 to 10% gradient SDS-PAGE under reducing conditions. Immunoprecipitated HMGCR was revealed with a rabbit anti-HMGCR Ab coupled to Alexa Fluor 680 and read on an Odyssey Li-Cor (Li-Cor Biosciences, Lincoln, NE, USA).

### Addressable laser bead immunoassay (ALBIA) for the detection and quantification of anti-HMGCR aAbs (ALBIA-HMCGR)

We previously described an ALBIA for anti-SRP antibodies (ALBIA-SRP) [16]. Here, to develop immunoassay for anti-HMGCR antibodies (ALBIA-HMGCR), a similar strategy was applied with some modifications. Five to 12 μg of recombinant HMGCR C-domain were coupled to 1.25 × 10^6^ fluorescent Bio-Plex^™^ COOH-microspheres (Bio-Rad, Hercules, CA, USA) with the Bio-Plex^™^ amine coupling kit (Bio-Rad) according to manufacturer’s protocol. After optimization to maximize the signal/noise ratio, the quantity of HMGCR protein per coupling reaction was set at 10 μg. After coupling, coated beads were either used immediately or stored at -20°C in the dark. When indicated, a human recombinant SRP or intrinsic factor protein (Diarect AG, Freiburg, Germany) was used in place of HMGCR for controls.

Immediately prior to use, HMGCR-coated beads were vigorously agitated for 30 s. Then, a 10 μl volume containing 1,250 beads was added to 100 μl of serum from patients or controls (diluted in Dulbecco's phosphate-buffered saline (DPBS) plus 1% fetal bovine serum) in Multiscreen 96-well plates (Millipore, Bedford, MA, USA). Plates were incubated for 2 h at room temperature in the dark on a plate shaker at 650 rpm. Blank (no serum, secondary antibody only), negative control (anti-HMGCR negative serum) and positive controls (highly positive human anti-HMGCR Ab serum or rabbit anti-HMGCR Abs with appropriate secondary antibody) were included in every assay. Beads were collected by filtration under vacuum and washed twice with 150 μl DPBS containing 0.1% Tween-20. Biotinylated mouse anti-human IgG- or isotype (IgG1, IgG2, IgG3 or IgG4)-specific secondary Ab (Southern Biotech, Birmingham, AL, USA) were added at 1/2,000 dilution and incubated for 1 h at room temperature under shaking. After washing, beads were incubated with 50 μl of streptavidin-R-phycoerythrin (Qiagen, Venlo, The Netherlands) at 1/1,000 dilution for 15 min. Finally, beads were resuspended in 100 μl of DPBS and mean fluorescence intensity (MFI) was determined on a Bio-Plex^™^ apparatus using the Bio-Plex^™^ Manager software 4.0 (Bio-Rad).

The analytical sensitivity of detection of ALBIA-HMGCR was determined by triplicate dosage of serial dilutions of rabbit anti-HMGCR antibodies. For each well, after subtraction of negative control MFI value, mean MFI and standard deviation (SD) were calculated.

The analytical specificity of detection of ALBIA-HMGCR was determined by using high-level human anti-HMGCR, anti-SRP or anti-intrinsic factor positive sera at 1/1,000 dilution. Sera were incubated with purified HMGCR, SRP or intrinsic factor beads for 1 h at room temperature and MFI was determined as described above.

Specific inhibition of ALBIA-HMGCR was performed using increasing concentrations of recombinant HMGCR protein, with 1/2,000 dilution of the anti-HMGCR serum used as 100% reference. Percent inhibition was calculated as (1 - (MFI pre-adsorbed serum/MFI serum)) × 100. Homologous and heterologous inhibition of ALBIA-HMGCR was further performed by pre-absorption of three anti-HMGCR, three anti-SRP or three anti-intrinsic factor positive sera with 100 μg/ml of recombinant HMGCR, SRP or intrinsic factor protein.

All patients' sera were initially assayed at a 1/500 dilution. The anti-HMGCR Ab levels were determined at a 1/D dilution using the following formula: (MFI^serum^/MFI^calibrator^) × (level of calibrator) × D/500. The calibrator is a human highly positive anti-HMGCR serum (the same throughout the study) whose level was arbitrarily set to 100 arbitrary units (AU)/mL). When sample MFI at 1/500 dilution was higher than 80% of calibrator MFI, further dilutions were performed and the first dilution yielding a MFI inferior to 80% of calibrator MFI was retained for calculation of level.

Reproducibility of ALBIA-HMGCR was determined using three independent anti-HMGCR Ab positive sera (low, medium and high level). Intra-assay variation was determined by measurements of the same samples within the same run. Inter-assay variation (assay to assay) was determined by measurements of the same samples in separate runs. Inter-assay variation (batch to batch) was determined by measurement of the same samples in the same run using different batches of coupled beads. Coefficients of variation (CV) were computed as SD/mean.

### Multiplex ALBIA for the simultaneous detection and quantification of anti-HMGCR and anti-SRP aAbs in NAM patients (ALBIA-NAM)

To detect simultaneously anti-SRP or anti-HMGCR aAbs from a single sample, we used two different beads harboring a special mixture of red/infrared dyes so that they are endowed with a specific spectral signature. Color codes of HMGCR- and SRP-coupled beads were number 55 and 28 (Bio-Rad), respectively. After coupling with HMGCR or SRP, 10 μl of each bead preparation were mixed and incubated with serum samples in the same conditions than described previously. Analytical specificity of ALBIA-NAM was further determined by analyzing sera from patients with anti-HMGCR or anti-SRP aAbs or from healthy controls or patients with different inflammatory/autoimmune conditions including RA, systemic sclerosis, SLE, DM, anti-tRNA synthetase aAb positive myositis and IBM, or polyclonal hypergammaglobulinemia. Levels of anti-SRP and anti-HMGCR sera were compared with those obtained with the corresponding monoplex ALBIA-HMGCR or ALBIA-SRP.

### HMGCR line immunoassay and immunofluorescence analysis

The Myositis Euroline 7 (IgG) line immunoassay was kindly provided by Euroimmun (Lübeck, Germany). It consists in a membrane strip coated with different antigens, including two different recombinant HMGCR proteins. The first consists of the HMGCR C-domain fused to GST and the second a human cDNA expressed in *Escherichia coli*. The assay was used according to the manufacturer’s instruction and the intensity of coloration was graded from one to four.

Indirect immunofluorescence was performed on HEp-2000™ cells (ImmunoConcepts, Sacramento, CA, USA) and Kallestad HEp-2 cells (Bio-Rad). Sera were tested at 1/80 screening dilution in PBS buffer, using a FITC-coupled antibody against human IgG.

### Statistical analysis

The normality of data distribution was analyzed using the D'Agostino and Pearson test, and the receiver operating characteristic (ROC) curve was determined using Prism software (GraphPad Software, La Jolla, CA, USA).

## Results

### Sensitivity and specificity of ALBIA-HMGCR

**Figure 1:**
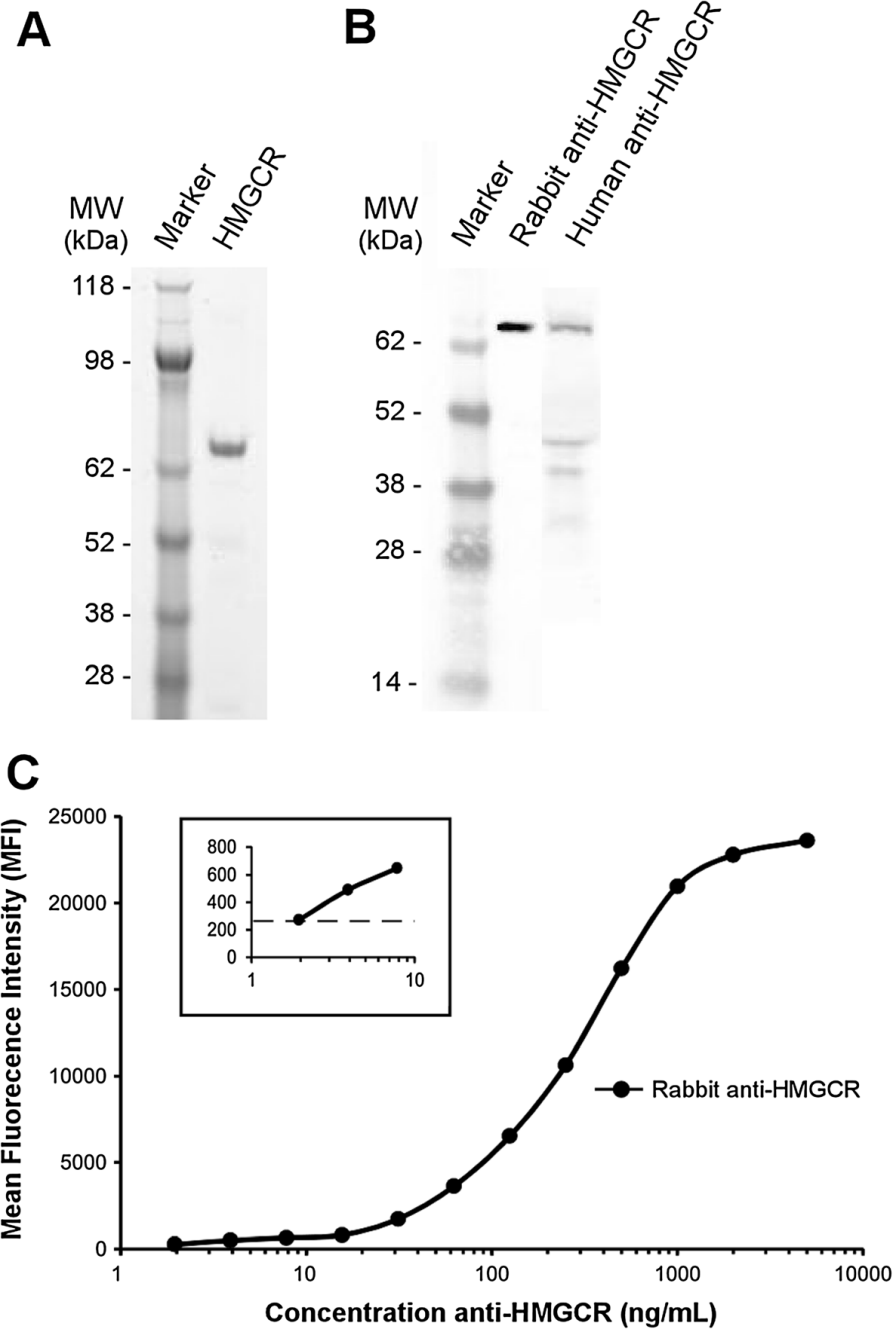
**Development of a quantitative assay for the detection of anti-HMGCR antibodies. (A)** SDS-PAGE analysis of the recombinant HMGCR catalytic C-domain after Coomassie blue staining. **(B)** Western blot analysis of this recombinant HMGCR using a specific rabbit anti-HMGCR antibody or a human anti-HMGCR-positive serum. **(C)** Addressable laser bead immunoassay (ALBIA-HMGCR) using serial dilutions of rabbit anti-HMGCR antibody. The mean fluorescence intensity (MFI) values are the mean of triplicate determinations. Standard deviation (SD) errors bars are not visible because of very low variability (coefficients of variation SD/mean lower than 1%, range (0.0025 to 0.7525)). Insert, higher magnification for the low anti-HMGCR antibody concentrations (horizontal dotted line depicts mean + 2 SD of 20 negative controls). HMGCR, 3-hydroxy-3-methylglutaryl coenzyme A reductase.

To allow quantitative analysis of anti-HMGCR aAbs in patients, we developed an addressable laser bead immunoassay (ALBIA-HMGCR) [16,23]. For this, we used as antigen the recombinant catalytic C-domain of the human HMGCR protein fused to GST. Purity of this protein was confirmed by Coomassie blue staining after SDS-PAGE, revealing a unique band (Figure 1A) that was specifically recognized by rabbit anti-HMGCR Abs or a human anti-HMGCR positive serum in Western blot (Figure 1B).

HMGCR was coupled through a covalent link to fluorescent beads and used to measure the levels of anti-HMGCR Abs. The development of ALBIA-HMGCR first required optimizing concentration of recombinant HMGCR protein used to coat beads, incubation times, washing steps and revelation with the secondary antibody (not shown, see Material and Methods for details). This yielded a method allowing detection of as low as 5 ng/mL anti-HMGCR Abs (Figure 1C), indicating a very good analytical sensitivity of the assay.

**Figure 2:**
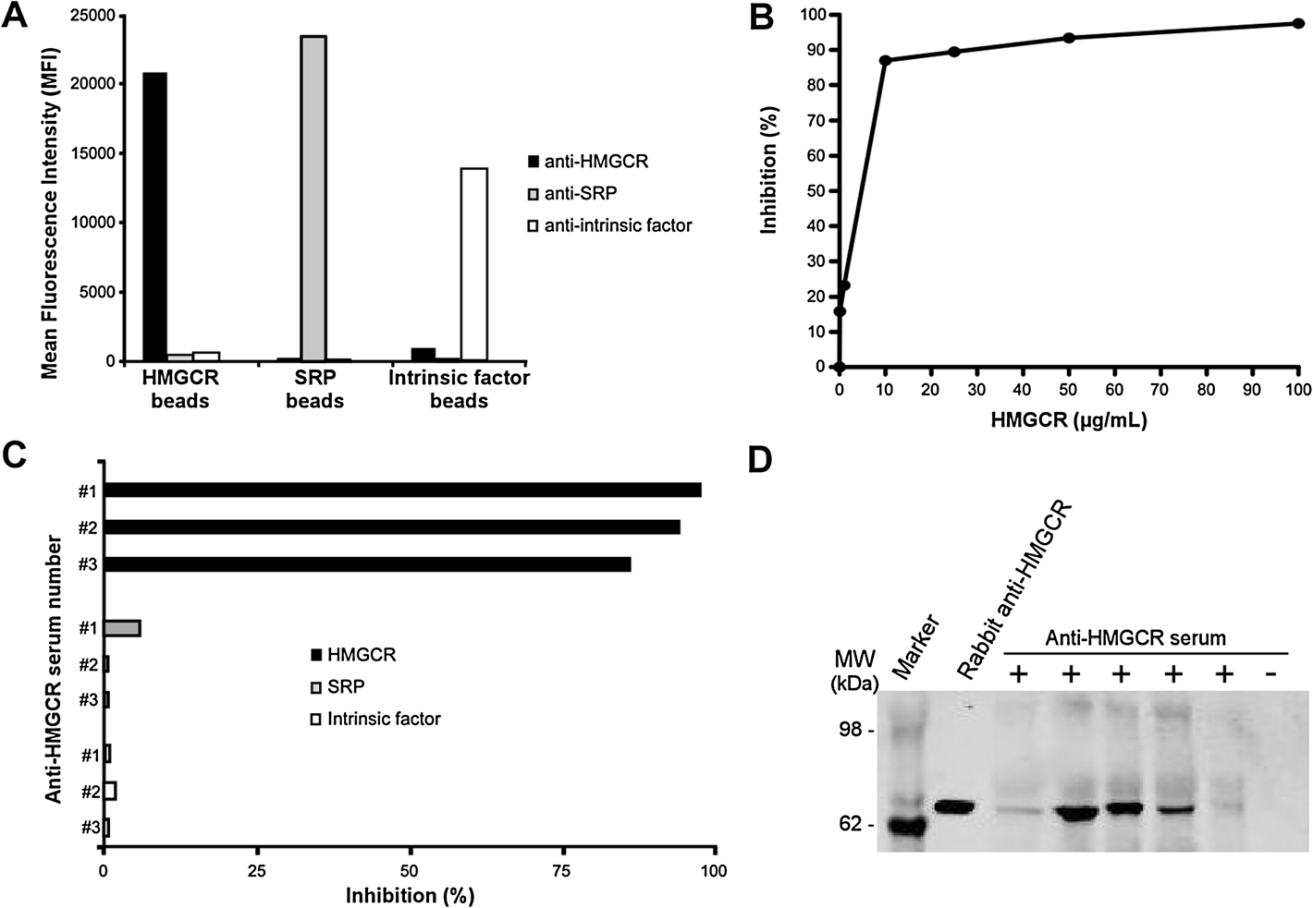
**Specificity of HMGCR-coated beads. (A)** HMGCR, signal recognition particle (SRP) and intrinsic factor-specific beads were coupled to the corresponding recombinant protein and analyzed by ALBIA using a human anti-HMGCR, anti-SRP or anti-intrinsic factor positive serum. **(B)** Dose-dependent inhibition of human anti-HMGCR antibodies binding to HMGCR-coated beads by free human recombinant HMGCR protein. Percent inhibition is given relative to the mean fluorescence intensity (MFI) value in the absence of free HMGCR. **(C)** Specific inhibition by homologous HMGCR but not heterologous proteins. Percent inhibition of three different human anti-HMGCR positive sera (referred to as #1, #2 and #3) by 100 μg/mL of free HMGCR, SRP or intrinsic factor proteins. **(D)** Immunoprecipitation of HMGCR protein by ALBIA-HMGCR positive or negative human sera. A rabbit anti-HMGCR polyclonal antibody was used as positive control. ALBIA, addressable laser bead immunoassay; HMGCR, 3-hydroxy-3-methylglutaryl coenzyme A reductase.

The specificity of HMGCR-coated beads was next evaluated. For this, HMGCR, SRP and intrinsic factor-specific beads were prepared and analyzed using a human anti-HMGCR, anti-SRP or anti-intrinsic factor positive serum. Only anti-HMGCR but not irrelevant anti-SRP or anti-intrinsic factor Ab-positive sera reacted with HMGCR beads (Figure 2A). Similarly, beads coated with SRP or intrinsic factor recombinant proteins that were produced and purified using the same process that for HMGCR protein reacted only with the corresponding aAb-positive serum.

Addition of free homologous protein, that is HMGCR, resulted in a dose-dependent inhibition of the HMGCR signal (Figure 2B) while no inhibition was observed when using a heterologous protein, that is SRP or intrinsic factor, at the concentration of 100 μg/mL (Figure 2C). To further confirm the antigenic specificity of the assay, five sera that scored positive for anti-HMGCR aAbs using ALBIA-HMGCR were used to immunoprecipitate the recombinant HMGCR protein. This yielded a positive result in 5/5 cases but not when using an irrelevant serum (Figure 2D).

### Determination of anti-HMGCR aAb levels

The method used for calculating anti-HMGCR levels is illustrated in an additional figure (see Additional file 1). The serum used in this example showed a saturating signal at the 1/500 screening dilution. A further 1/4,000 dilution was retained to calculate the level by reference to a highly positive anti-HMGCR serum used as calibrator whose level was arbitrarily set to 100 AU/mL.

### Reproducibility and repeatability of the ALBIA-HMGCR

Reproducibility and repeatability of the test were determined by calculating both intra- and inter-assay variation for sera with high, medium and low anti-HMGCR levels. The intra-assay coefficients of variation (CV) ranged from 5% for high/medium levels to 13% for low levels, as indicated in an additional table (see Additional file 2). Similarly, the mean CV for inter-assay variation, as determined from plate-to-plate or batch-to-batch variations ranged from 9 to 11%, and 2.8 to 5.6%, respectively. Together, these data indicate a good reproducibility and repeatability of the ALBIA-HMGCR assay.

### Validation of ALBIA-HMGCR

**Figure 3:**
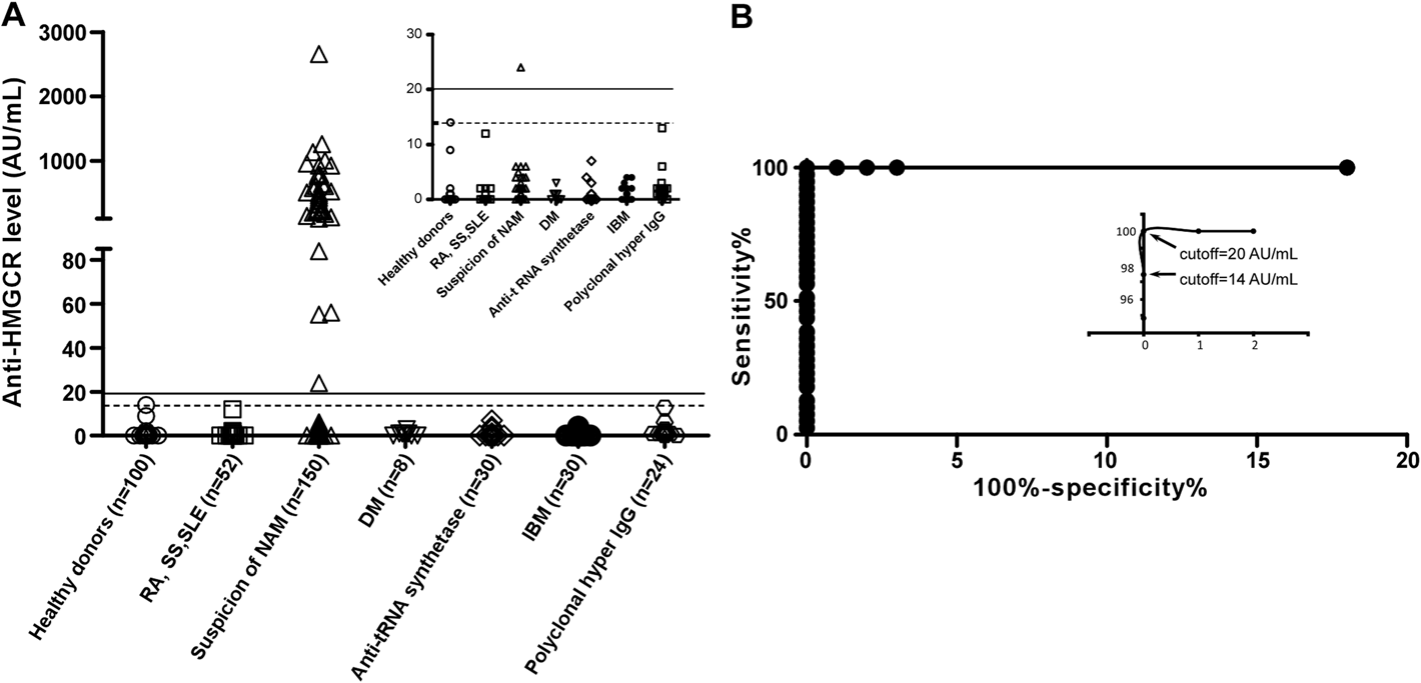
**Validation of ALBIA-HMGCR and determination of anti-HMGCR levels in patients with suspicion of NAM. (A)** A positivity cutoff of the assay was first determined as the 99^th^ percentile (14 arbitrary units (AU)/mL) of the healthy donors’ distribution (open circles) and depicted by a dotted line. Sera from patients with different inflammatory/autoimmune conditions including rheumatoid arthritis (RA), systemic sclerosis (SS), systemic lupus erythematosus (SLE), dermatomyositis (DM), anti-tRNA synthetase-positive myositis or inclusion body myositis (IBM), as well as patients with polyclonal hypergammaglobulinemia were assayed and revealed all negative. Insert is a magnification for low values. Thirty-seven out of 150 (24%) of serum samples from patients with suspicion of NAM scored positive using ALBIA-HMGCR. **(B)** Receiver operating characteristic (ROC) analysis was performed by comparing the 37 anti-HMGCR positive serum samples to those from healthy donors. Optimal results were obtained with a cutoff of 20 AU/mL, which was indicated as a plain line. ALBIA, addressable laser bead immunoassay; HMGCR, 3-hydroxy-3-methylglutaryl coenzyme A reductase; NAM, necrotizing autoimmune myopathies.

We used ALBIA-HMGCR to screen serum samples for the presence of anti-HMGCR aAbs. First, a threshold of positivity was calculated using 100 control sera from healthy blood donors. The values obtained with control sera did not follow a normal distribution (*P* <0.0001), their median was 0 AU/mL and the 99^th^ percentile was 14 AU/mL. At a cutoff of 14 AU/mL, 99/100 control sera scored negative (Figure 3A).

Next, we analyzed sera from 150 patients with suspicion of NAM. Of these, 37 patients (24%) were anti-HMGCR positive, with levels ranging from 24 to 2.656 AU/mL. ROC curve analysis revealed a specificity of 100% and a sensitivity of 100% when the cutoff was set at 20 AU/ml (Figure 3B). Patients with different inflammatory/autoimmune conditions including RA, systemic sclerosis, SLE, DM, anti-tRNA synthetase Ab positive myositis or IBM, as well as patients with polyclonal hypergammaglobulinemia all scored negative (Figure 3A). Anti-HMGCR positive and 16 negative sera from healthy donors were further tested by a recently developed line immunoassay. All 37 ALBIA-HMGCR positive sera were positive using this assay while 16/16 controls were negative (not shown). Yet, there was no correlation between band intensity and ALBIA-HMGCR level.

### Immunofluorescence aspect on HEp-2 cells

**Figure 4:**
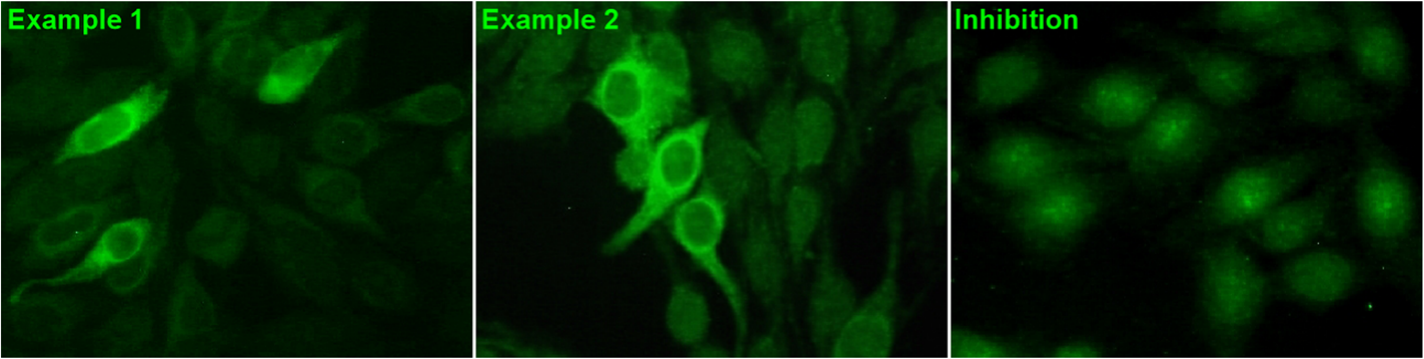
**Aspect of anti-HMGCR autoantibodies in indirect immunofluorescence on HEp-2 cells.** Classical indirect immunofluorescence assay using the HEp-2000^™^ line as cellular substrate. After incubation of serum at a 1/80 dilution, immunoreactivity was revealed by a FITC-labeled anti-human immunoglobulin (Ig)G secondary antibody. Example from two representative anti-HMGCR positive patients out of 20/33 sera with this immunofluorescence pattern shows a finely granular cytoplasmic staining with perinuclear reinforcement (left and middle). Inhibition by free HMGCR protein before immunofluorescence (right): the serum from example 2 was pre-incubated with free HMCGR before immunofluorescence assay. This yielded an extinction of immunoreactivity. HMGCR, 3-hydroxy-3-methylglutaryl coenzyme A reductase.

Sixty-one percent (20/33) of ALBIA-HMGCR positive sera displayed a finely granular cytoplasmic pattern on a minority (3% or less) of HEp-2000® cells (Figure 4). This immunoreactivity could be inhibited by previous incubation with free HMGCR. Using Kallestad HEp-2 cells, only 30% of the ALBIA-HMGCR positive sera yielded this aspect (not shown).

### IgG isotypes of anti-HMGCR Abs

Fifteen ALBIA-HMGCR positive sera were tested for anti-HMGCR IgG subclasses. Only IgG1 were represented among anti-HMGCR aAbs in this series (not shown). IgG2, IgG3 or IgG4 subclasses were never found, neither as a single isotype nor in association with IgG1.

### Exploring necrotizing autoimmune myopathies with multiplex ALBIA-NAM

**Figure 5:**
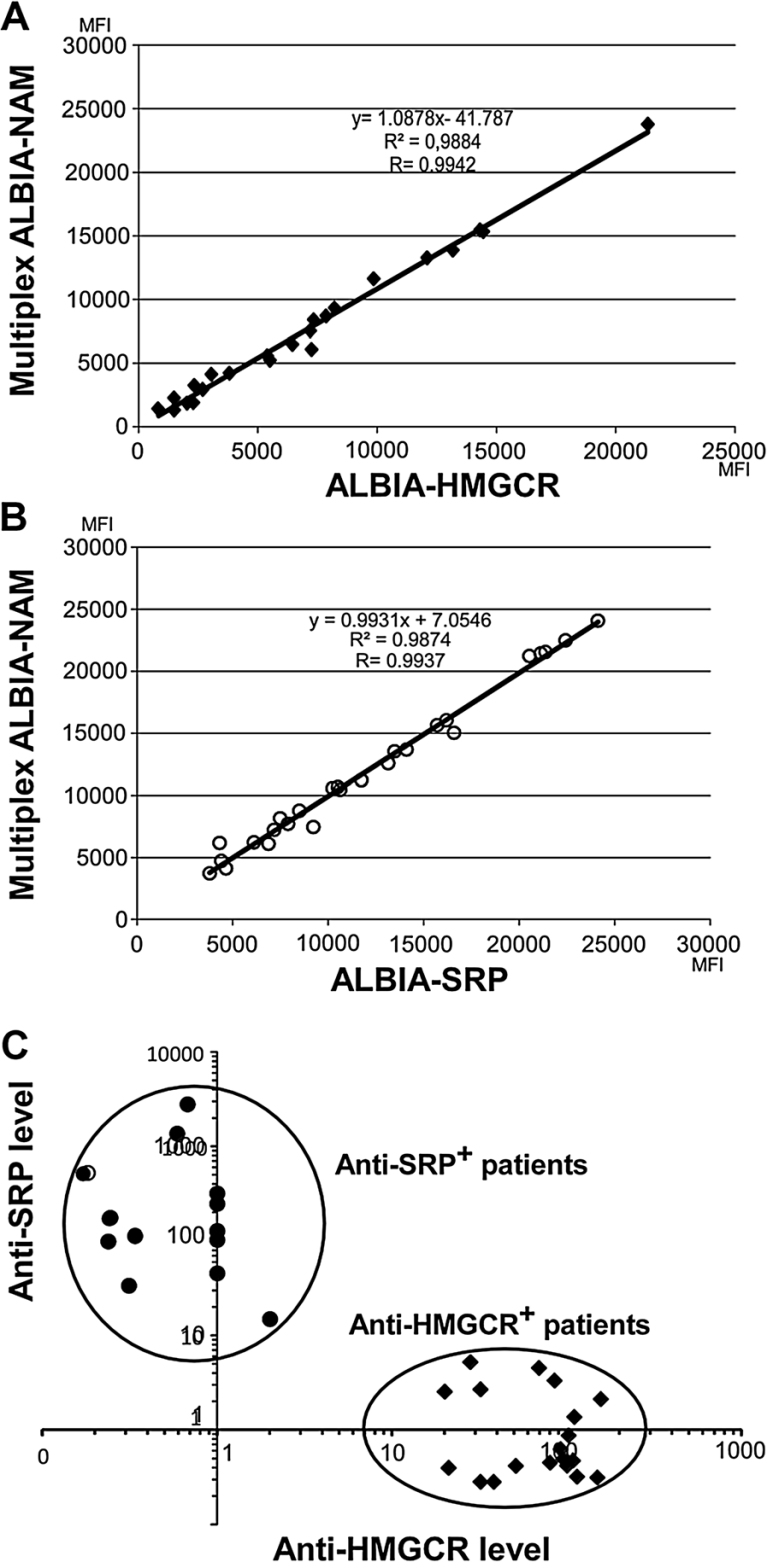
**Comparison of monoplex ALBIA-HMGCR and ALBIA-SRP to multiplex ALBIA-NAM.** Serum from anti-HMGCR positive (n = 26) or anti-SRP positive (n = 25) patients were compared. **(A)** Correlation of the signals generated by ALBIA-NAM versus ALBIA-HMGCR. Mean fluorescence intensity (MFI) **(B)** Correlation of the signals generated by ALBIA-NAM versus ALBIA-SRP. **(C)** ALBIA-NAM discriminates anti-SRP positive from anti-HMGCR positive patients in a single assay. ALBIA, addressable laser bead immunoassay; HMGCR, 3-hydroxy-3-methylglutaryl coenzyme A reductase; SRP, signal recognition particle.

In order to simultaneously quantify anti-HMGCR and anti-SRP aAbs, we next developed a multiplex assay (ALBIA-NAM) and compared its results to that of monoplex ALBIA-HMGCR and ALBIA-SRP [16]. For this, we assayed 26 anti-HMGCR positive patients from the present series plus 25 anti-SRP positive sera that were described previously [16]. Results from both assays were compared by linear regression and showed excellent correlation, with *R* values of 0.9942 and 0.9937 for ALBIA-HMGCR versus ALBIA-NAM (Figure 5A) and ALBIA-SRP versus ALBIA-NAM (Figure 5B), respectively.

ALBIA-NAM revealed able to perfectly discriminate the two populations of NAM patients, that is those with anti-HMGCR from those with anti-SRP aAbs (Figure 5C) and was negative for all other tested conditions, that is patients with different inflammatory/autoimmune conditions, DM, anti-tRNA synthetase Ab positive myositis or IBM, as well as patients with polyclonal hypergammaglobulinemia (see Additional file 3). The sensitivity of monoplex and multiplex assays was equivalent. No NAM patient was positive for both aAbs.

### Characteristics of anti-HMGCR positive patients

Characteristics of anti-HMGCR positive patients are summarized in Table 1. These patients presented with proximal muscle weakness (92%), had elevated (mean >6,000 IU/L) CK levels and 60% had not taken statins. Muscle biopsies always showed regenerating and necrotic muscle fibers, with occasionally (28%) perivascular inflammatory infiltrates.

**Table 1:** Clinical characteristics of anti-HMGCR positive patients (n = 37)

**Age (mean ± SD):**	**44 ± 19 years**
Sex (M/F):	12/25
Muscle weakness:	34/37 (92%)
Creatine kinase level (mean ± SD):	6,974 ± 4,970 IU/L
Statin exposure:	15/37 (40%)

HMGCR, 3-hydroxy-3-methylglutaryl coenzyme A reductase; SD, standard deviation.

## Discussion

This report describes the first immunoassay which can detect and quantify simultaneously anti-HMGCR and anti-SRP aAbs in patients with NAM, a recently identified, severe form of inflammatory myopathy with important muscle necrosis/regeneration and little inflammation. Up to recently, the first aAb sought for in NAM patients was directed against SRP, a protein complex that guides the translocation of growing polypeptides into the endoplasmic reticulum during protein synthesis. Currently, the detection of anti-SRP aAbs may be suspected upon result of an indirect immunofluorescence test and the diagnosis must be confirmed by a specific assay, for example dot-blot. Recently, we described a new immunoassay, that is ALBIA-SRP, to quantify anti-SRP aAbs and investigated the relationship between serum CK levels, muscle strength and anti-SRP levels [16]. Results revealed a significant correlation between the degree of myolysis and the level of anti-SRP aAbs, suggesting that these aAbs may be pathogenic. In the present study, we applied a similar strategy to provide a new immunoassay for anti-HMGCR aAbs, and also validated a multiplex immunoassay to detect and quantify both anti-HMGCR and anti-SRP aAbs using a single biological test.

Whereas it cannot be excluded that some other epitopes might exist, a mapping study showed that anti-HMGCR aAbs recognize the catalytic C-domain of HMGCR, but do not react with the N-terminal domain of the protein [15]. Hence, beads were coated with a recombinant HMGCR C-domain protein. Rabbit anti-HMGCR Abs that were produced after immunization with a polypeptide corresponding to residues 550 to 650 of the human HMGCR C-domain could recognize the recombinant protein used herein for performing Western blot (Figure 1B) and ALBIA-HMGCR (Figure 1C). These results demonstrate the accessibility of recombinant HMGCR C-domain epitopes to anti-HMGCR Abs. The reactivity of the human anti-HMGCR positive serum against some smaller bands (approximately 40 to 45 kDa) in the Western blot (Figure 1B) presumably indicates that the recombinant HMGCR antigen has some breakdown products that are not recognized by the rabbit serum that was obtained by immunization with only a fragment of HMCGR C-domain.

ALBIA-HMGCR detected anti-HMGCR aAbs in 37 individuals (24%) among a series of 150 patients with suspicion of NAM (Figure 3A). A perfect concordance with a recently developed line immunoassay was found. Yet, there was no correlation between band intensity in this assay and ALBIA-HMGCR level. Further, ALBIA-HMGCR remained negative when tested against sera from patients with different inflammatory/autoimmune conditions including RA, systemic sclerosis, SLE, DM, anti-tRNA synthetase-positive myositis or IBM, or in patients with polyclonal hypergammaglobulinemia (Figure 3A). ROC analysis revealed a cutoff for positivity of 20 AU/mL, providing a high diagnostic value (Figure 3B).

Anti-HMGCR IgG subclass distribution, which had remained unknown until now, was determined. Contrary to what was observed for anti-SRP positive patients in whom IgG1 and IgG4 were principally observed while all four subclasses could possibly be found depending on the patient [16], or to other autoimmune diseases such as myasthenia gravis, membranous glomerulonephritis or pemphigus, in which both IgG1 and IgG4 isotypes have been found [24-26], the only subclass detected in anti-HMGCR positive patients was IgG1 in the present study.

Using immunofluoresence on HEp-2 cells, 30 to 61% of anti-HMGCR positive sera exhibited a finely granular cytoplasmic pattern with perinuclear reinforcement in a minority of cells (Figure 4). Yet, the cellular substrates used for immunofluorescence appeared heterogeneous in terms of HMGCR expression levels and some commercial sources may provide HEp-2 cells less prone to allow detecting anti-HMGCR aAbs than others.

Christopher-Stine *et al*. found a 7% prevalence of anti-HMGCR aAbs (as initially determined by a 100 to 200 kDa reactivity after immunoprecipitation) within a series of 225 patients with proximal muscle weakness, elevated CK, myopathic findings on electromyography, muscle edema on magnetic resonance imaging and/or features of myopathy on muscle biopsy [14]. This prevalence was higher in a subgroup of 38 patients with necrotizing myopathy among whom 16 (42%) were found positive for anti-HMGCR aAbs.

In a general population of statin users, with or without self-limited musculoskeletal side effects, the prevalence of anti-HMGCR aAbs is extremely low [27]. In contrast, Mammen *et al*. reported that 67% of anti-HMGCR positive NAM patients had been exposed to statins [14,15]. Here, we found that 40% of our anti-HMGCR positive patients had taken statins. Therefore, although anti-HMGCR aAbs are directed against the statins target itself, the diagnosis of anti-HMGCR positive NAM should not be excluded *a priori* in patients who have not been exposed to statins.

An advantage of the ALBIA methodology is to allow the concomitant detection of several aAbs in a single assay from a unique serum sample, therefore reducing number of biological tests, labor and time necessary between diagnosis and therapy. In this study, we multiplexed ALBIA-HMGCR and ALBIA-SRP to provide the multiplex ALBIA-NAM. The performance of ALBIA-NAM was compared to that of the parental monoplex immunoassays. Linear regression analysis showed an excellent correlation between monoplex and multiplex assays (Figure 5A,B). None of the sera found positive by monoplex assays scored negative when using ALBIA-NAM and *vice versa*. Furthermore, all anti-HMGCR positive sera were negative for anti-SRP aAbs, suggesting that patients with anti-SRP or anti-HMGCR aAb might belong to different NAM subgroups (Figure 5C). Yet, a fraction of NAM patients remains without any known aAb, suggesting that new auto-antigenic specificities are still to be discovered.

Together, the results of this study show that multiplex ALBIA-NAM allows efficient detection and quantification of anti-HMGCR or anti-SRP aAbs in patients with suspicion of NAM and that the diagnosis of anti-HMGCR positive NAM should not be excluded *a priori* in patients who have not been exposed to statins.

## Conclusions

Diagnosis and clinical studies of necrotizing autoimmune myopathies (NAM) have been hampered by the paucity of biological assays for the assessment of specific biomarkers. Some NAM patients have autoantibodies (aAbs) against the statins drug target, that is 3-hydroxy-3-methyl-glutaryl-coenzyme A reductase (HMGCR). Here, we developed a new immunoassay allowing efficient detection and quantification of anti-HMGCR aAbs in patients with suspicion of NAM. In a series of 150 patients, 24% were found positive for anti-HMGCR aAbs. Only 40% of anti-HMGCR-positive patients had been exposed to statins. Hence, lack of statin exposure should not eliminate the diagnosis of anti-HMGCR-positive NAM. This assay was multiplexed with an anti-signal recognition particle (SRP) aAb immunoassay, facilitating the diagnosis of NAM using a single assay.

Using indirect immunofluorescence, some anti-HMGCR positive sera exhibited a finely granular cytoplasmic pattern with perinuclear reinforcement in a minority of cells. Necrotizing myopathies may also encompass some forms of muscular dystrophy. Together, the present assays will be helpful for the diagnosis of necrotizing myopathies since a positive result allows ascribing patients to an autoimmune form.

## Abbreviations

aAbs: autoantibodies; ALBIA: addressable laser bead immunoassay; AU: arbitrary units; CK: creatine kinase; CV: coefficient of variation; DM: dermatomyositis; DPBS: Dulbecco's phosphate-buffered saline; GST: glutathione S-transferase; HMGCR: 3-hydroxy-3-methylglutaryl coenzyme A reductase; IBM: inclusion body myositis; Ig: immunoglobulin; MFI: mean fluorescence intensity; NAM: necrotizing autoimmune myopathies; RA: rheumatoid arthritis; ROC: receiver operating characteristic; SD: standard deviation; SLE: systemic lupus erythematosus; SRP: signal recognition particle.

## Competing interests

The authors declare that they have no competing interests.

## Authors’ contributions

LD, OBe, OBo and FJ designed the study. LD and OBo developed the immunoassays. LD, FJ, YA, JLC, JM, LM, OBe, AM, OH, BBM, IKP, ECS, BE, AT, JS and IM participated to acquisition and interpretation of data. LD, OBe, OBo, FJ, YA, JLC and LM analyzed and interpreted the data. LD and OBo wrote the first draft of the manuscript. OBo had full access to all of the data in the study and takes responsibility for the integrity of the data and the accuracy of the data analysis. All authors read and approved the final manuscript.

## Supplementary Material

Additional file 1 **Determination of the level of anti-HMGCR aAbs.** Anti-HMGCR levels were determined by reference to the MFI value, in the same assay, of a calibrator that is, a highly positive anti-HMGCR + serum whose level was arbitrarily set to 100 AU/mL. The assay was first performed using a 1/500 screening dilution of the serum. In this example, the sample’s MFI at a 1/500 dilution was higher than 80% of the calibrator’s MFI. Thus, further dilutions were performed and the first dilution yielding a MFI inferior to 80% of the calibrator MFI was retained for calculation, yielding a 428 AU/mL anti-HMGCR level. aAbs, autoantibodies; AU, arbitrary units; HMGCR, 3-hydroxy-3-methylglutaryl-coenzyme A reductase; MFI, mean fluorescence intensity. Click here for file

Additional file 2 **Reproducibility of ALBIA-HMGCR.** Intra- and inter-assay reproducibility as determined by the coefficients of variation (CV%) for repeated measures of high, medium and low anti-HMGCR level samples. ALBIA, addressable laser bead immunoassay; HMGCR, 3-hydroxy-3-methylglutaryl-coenzyme A reductase. Click here for file

Additional file 3 **Validation of ALBIA-NAM.** A positivity cutoff of the multiplex assay (dotted line) was determined as the 99th percentile of the healthy donors’ distribution (open circles) for both **(A)** anti-SRP (9 AU/mL) and **(B)** anti-HMGCR aAbs (8 AU/mL). Sera from patients with different inflammatory/autoimmune conditions including rheumatoid arthritis (RA), systemic sclerosis (SS), systemic lupus erythematosus (SLE), dermatomyositis (DM), anti-tRNA synthetase aAb-positive myositis or inclusion body myositis (IBM), as well as patients with polyclonal hypergammaglobulinemia were assayed. aAbs, autoantibodies; ALBIA, addressable laser bead immunoassay; AU, arbitrary units; HMGCR, 3-hydroxy-3-methylglutaryl coenzyme A reductase; NAM, necrotizing autoimmune myopathies; SRP, signal recognition particle. Click here for file

## Collaborators (French Myositis Network)

Pierre Kieffer, Tanya Stojkovic, Alexandra Nadaj-Pakleza, Bénédicte Giroux-Leprieur, Claire Laroche, Pascal Laforet, Anthony Béhin, Patrice Cacoub, Mamy Ralazamahaleo, Xavier Ferrer, Marielle Roux, Anne-Laure Bedat-Millet, Jean-Michel Ristori, Anne-Sophie Deleplancque, Julie Cosserat, Aude Rigolet, Laurent Arnaud, Laurent Servais, Chafika Morati, Nathalie Costedoat, Olivier Fain, Bernard Grosbois, Raphael De Paz, Noémie Le Gouellec, Guillaume Gondran, François Maurier, François-Jérôme Authier, Romain Ghérardi, Dimitri Renard, Antoine Laudat, Cédric Ménard, Isabelle Bahon, Claude Lambert, Léonard Feasson, Jean-Claude Antoine, Alfred Mahr.

## References

[B1] Dalakas MC Hohlfeld R Polymyositis and dermatomyositis Lancet 2003 16 971 982 10.1016/S0140-6736(03)14368-1 14511932

[B2] Bronner IM Hoogendijk JE Wintzen AR van der Meulen MF Linssen WH Wokke JH de Visser M Necrotising myopathy, an unusual presentation of a steroid-responsive myopathy J Neurol 2003 16 480 485 10.1007/s00415-003-1027-y 12700915

[B3] van der Meulen MF Bronner IM Hoogendijk JE Burger H van Venrooij WJ Voskuyl AE Dinant HJ Linssen WH Wokke JH de Visser M Polymyositis: an overdiagnosed entity Neurology 2003 16 316 321 10.1212/WNL.61.3.316 12913190

[B4] Sadeh M Dabby R Steroid-responsive myopathy: immune-mediated necrotizing myopathy or polymyositis without inflammation? J Clin Neuromuscul Dis 2008 16 341 344 10.1097/CND.0b013e31815e5d4a 18344715

[B5] Emslie-Smith AM Engel AG Necrotizing myopathy with pipestem capillaries, microvascular deposition of the complement membrane attack complex (MAC), and minimal cellular infiltration Neurology 1991 16 936 939 10.1212/WNL.41.6.936 2046947

[B6] Hoogendijk JE Amato AA Lecky BR Choy EH Lundberg IE Rose MR Vencovsky J de Visser M Hughes RA 119th ENMC international workshop: trial design in adult idiopathic inflammatory myopathies, with the exception of inclusion body myositis, 10–12 October 2003, Naarden, The Netherlands Neuromuscul Disord 2004 16 337 345 10.1016/j.nmd.2004.02.006 15099594

[B7] Brouwer R Hengstman GJ Vree Egberts W Ehrfeld H Bozic B Ghirardello A Grondal G Hietarinta M Isenberg D Kalden JR Lundberg I Moutsopoulos H Roux-Lombard P Vencovsky J Wikman A Seelig HP van Engelen BG van Venrooij WJ Autoantibody profiles in the sera of European patients with myositis Ann Rheum Dis 2001 16 116 123 10.1136/ard.60.2.116 11156543 PMC1753477

[B8] Hengstman GJ ter Laak HJ Vree Egberts WT Lundberg IE Moutsopoulos HM Vencovsky J Doria A Mosca M van Venrooij WJ van Engelen BG Anti-signal recognition particle autoantibodies: marker of a necrotising myopathy Ann Rheum Dis 2006 16 1635 1638 10.1136/ard.2006.052191 16679430 PMC1798474

[B9] Targoff IN Johnson AE Miller FW Antibody to signal recognition particle in polymyositis Arthritis Rheum 1990 16 1361 1370 10.1002/art.1780330908 2403400

[B10] Troyanov Y Targoff IN Tremblay JL Goulet JR Raymond Y Senecal JL Novel classification of idiopathic inflammatory myopathies based on overlap syndrome features and autoantibodies: analysis of 100 French Canadian patients Medicine (Baltimore) 2005 16 231 249 10.1097/01.md.0000173991.74008.b0 16010208

[B11] Kao AH Lacomis D Lucas M Fertig N Oddis CV Anti-signal recognition particle autoantibody in patients with and patients without idiopathic inflammatory myopathy Arthritis Rheum 2004 16 209 215 10.1002/art.11484 14730618

[B12] Miller T Al-Lozi MT Lopate G Pestronk A Myopathy with antibodies to the signal recognition particle: clinical and pathological features J Neurol Neurosurg Psychiatry 2002 16 420 428 10.1136/jnnp.73.4.420 12235311 PMC1738058

[B13] Needham M Fabian V Knezevic W Panegyres P Zilko P Mastaglia FL Progressive myopathy with up-regulation of MHC-I associated with statin therapy Neuromuscul Disord 2007 16 194 200 10.1016/j.nmd.2006.10.007 17241784

[B14] Christopher-Stine L Casciola-Rosen LA Hong G Chung T Corse AM Mammen AL A novel autoantibody recognizing 200-kd and 100-kd proteins is associated with an immune-mediated necrotizing myopathy Arthritis Rheum 2010 16 2757 2766 10.1002/art.27572 20496415 PMC3026777

[B15] Mammen AL Chung T Christopher-Stine L Rosen P Rosen A Doering KR Casciola-Rosen LA Autoantibodies against 3-hydroxy-3-methylglutaryl-coenzyme A reductase in patients with statin-associated autoimmune myopathy Arthritis Rheum 2011 16 713 721 10.1002/art.30156 21360500 PMC3335400

[B16] Benveniste O Drouot L Jouen F Charuel JL Bloch-Queyrat C Behin A Amoura Z Marie I Guiguet M Eymard B Gilbert D Tron F Herson S Musset L Boyer O Correlation of anti-signal recognition particle autoantibody levels with creatine kinase activity in patients with necrotizing myopathy Arthritis Rheum 2011 16 1961 1971 10.1002/art.30344 21400483

[B17] Werner JL Christopher-Stine L Ghazarian SR Pak KS Kus JE Daya NR Lloyd TE Mammen AL Antibody levels correlate with creatine kinase levels and strength in anti-3-hydroxy-3-methylglutaryl-coenzyme A reductase-associated autoimmune myopathy Arthritis Rheum 2012 16 4087 4093 10.1002/art.34673 22933019 PMC3510338

[B18] Tan EM Cohen AS Fries JF Masi AT McShane DJ Rothfield NF Schaller JG Talal N Winchester RJ The 1982 revised criteria for the classification of systemic lupus erythematosus Arthritis Rheum 1982 16 1271 1277 10.1002/art.1780251101 7138600

[B19] Arnett FC Edworthy SM Bloch DA McShane DJ Fries JF Cooper NS Healey LA Kaplan SR Liang MH Luthra HS Medsger TA Jr Mitchell DM Neustadt DH Pinals RS Schaller JG Sharp JT Wilder RL Hunder GG The American Rheumatism Association 1987 revised criteria for the classification of rheumatoid arthritis Arthritis Rheum 1988 16 315 324 10.1002/art.1780310302 3358796

[B20] Vitali C Bombardieri S Jonsson R Moutsopoulos HM Alexander EL Carsons SE Daniels TE Fox PC Fox RI Kassan SS Pillemer SR Talal N Weisman MH Classification criteria for Sjogren's syndrome: a revised version of the European criteria proposed by the American-European Consensus Group Ann Rheum Dis 2002 16 554 558 10.1136/ard.61.6.554 12006334 PMC1754137

[B21] Bohan A Peter JB Polymyositis and dermatomyositis (first of two parts) N Engl J Med 1975 16 344 347 10.1056/NEJM197502132920706 1090839

[B22] Griggs RC Askanas V DiMauro S Engel A Karpati G Mendell JR Rowland LP Inclusion body myositis and myopathies Ann Neurol 1995 16 705 713 10.1002/ana.410380504 7486861

[B23] Fritzler MJ Behmanesh F Fritzler ML Analysis of human sera that are polyreactive in an addressable laser bead immunoassay Clin Immunol 2006 16 349 356 10.1016/j.clim.2006.03.007 16644287

[B24] Makker SP Tramontano A Idiopathic membranous nephropathy: an autoimmune disease Semin Nephrol 2011 16 333 340 10.1016/j.semnephrol.2011.06.004 21839366 PMC3156416

[B25] Shigemoto K Myasthenia gravis induced by autoantibodies against MuSK Acta Myol 2007 16 185 191 18646570 PMC2949310

[B26] Sitaru C Mihai S Zillikens D The relevance of the IgG subclass of autoantibodies for blister induction in autoimmune bullous skin diseases Arch Dermatol Res 2007 16 1 8 10.1007/s00403-007-0734-0 17277959 PMC1839867

[B27] Mammen AL Pak K Williams EK Brisson D Coresh J Selvin E Gaudet D Rarity of anti-3-hydroxy-3-methylglutaryl-coenzyme A reductase antibodies in statin users, including those with self-limited musculoskeletal side effects Arthritis Care Res (Hoboken) 2012 16 269 272 10.1002/acr.20662 21972203 PMC3415973

