# Supplementary material for: Exploring necrotizing autoimmune myopathies with a novel immunoassay for anti-3-hydroxy-3-methyl-glutaryl-CoA reductase autoantibodies
Source: Arthritis Res Ther. 2014 Feb 3;16(1):R39. doi: 10.1186/ar4468 (PMC3979083; doi:10.1186/ar4468)
Supplement: Additional file 1 — Determination of the level of anti-HMGCR aAbs. Anti-HMGCR levels were determined by reference to the MFI value, in the same assay, of a calibrator that is, a highly positive anti-HMGCR + serum whose level was arbitrarily set to 100 AU/mL. The assay was first performed using a 1/500 screening dilution of the serum. In this example, the sample’s MFI at a 1/500 dilution was higher than 80% of the calibrator’s MFI. Thus, further dilutions were performed and the first dilution yielding a MFI inferior to 80% of the calibrator MFI was retained for calculation, yielding a 428 AU/mL anti-HMGCR level. aAbs, autoantibodies; AU, arbitrary units; HMGCR, 3-hydroxy-3-methylglutaryl-coenzyme A reductase; MFI, mean fluorescence intensity. [file ar4468-S1.pdf]

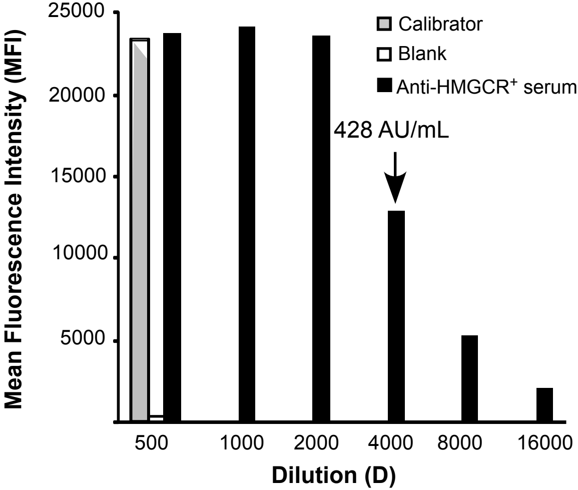

$$\text{Level (AU/mL)} = \left[\frac{\text{MFI}^{\text{serum}}}{\text{MFI}^{\text{calibrator}}}\right] \times [\text{cal}] \times \frac{D}{500}$$

$$[\text{cal}] = 100 \text{ AU/mL}$$

$$\text{Example: } \left[\frac{12700}{23700}\right] \times 100 \times \frac{4000}{500} = 428 \text{ AU/mL}$$

Additional file 1 Determination of the level of anti-HMGCR aAbs. Anti-HMGCR levels were determined by reference to the MFI value, in the same assay, of a calibrator i.e., a highly positive anti-HMGCR^{+} serum whose level was arbitrarily set to 100 AU/mL. The assay was first performed using a 1/500 screening dilution of the serum. In this example, the sample's MFI at a 1/500 dilution was higher than 80% of the calibrator's MFI Thus, further dilutions were performed and the first dilution yielding a MFI inferior to 80% of calibrator MFI was retained for calculation, yielding a 428 AU/mL anti-HMGCR level.
