# Supplementary material for: Exploring necrotizing autoimmune myopathies with a novel immunoassay for anti-3-hydroxy-3-methyl-glutaryl-CoA reductase autoantibodies
Source: Arthritis Res Ther. 2014 Feb 3;16(1):R39. doi: 10.1186/ar4468 (PMC3979083; doi:10.1186/ar4468)
Supplement: Additional file 2 — Reproducibility of ALBIA-HMGCR. Intra- and inter-assay reproducibility as determined by the coefficients of variation (CV%) for repeated measures of high, medium and low anti-HMGCR level samples. ALBIA, addressable laser bead immunoassay; HMGCR, 3-hydroxy-3-methylglutaryl-coenzyme A reductase. [file ar4468-S2.pdf]

Additional file 2

Reproducibility of ALBIA-HMGCR

Serum number	Anti-HMGCR level (AU/mL)	Intra-assay variation (within plate) %CV (n)	Inter-assay variation (assay to assay) %CV (n)	Inter-assay variation (batch to batch) %CV (n)
# 1	455 (high)	5 (25)	9 (30)	2.8 (2)
# 2	223 (medium)	5 (25)	11 (30)	4.5 (2)
# 3	29 (low)	13 (25)	10 (30)	5.7 (2)
