# Supplementary material for: Exploring necrotizing autoimmune myopathies with a novel immunoassay for anti-3-hydroxy-3-methyl-glutaryl-CoA reductase autoantibodies
Source: Arthritis Res Ther. 2014 Feb 3;16(1):R39. doi: 10.1186/ar4468 (PMC3979083; doi:10.1186/ar4468)
Supplement: Additional file 3 — Validation of ALBIA-NAM. A positivity cutoff of the multiplex assay (dotted line) was determined as the 99th percentile of the healthy donors’ distribution (open circles) for both (A) anti-SRP (9 AU/mL) and (B) anti-HMGCR aAbs (8 AU/mL). Sera from patients with different inflammatory/autoimmune conditions including rheumatoid arthritis (RA), systemic sclerosis (SS), systemic lupus erythematosus (SLE), dermatomyositis (DM), anti-tRNA synthetase aAb-positive myositis or inclusion body myositis (IBM), as well as patients with polyclonal hypergammaglobulinemia were assayed. aAbs, autoantibodies; ALBIA, addressable laser bead immunoassay; AU, arbitrary units; HMGCR, 3-hydroxy-3-methylglutaryl coenzyme A reductase; NAM, necrotizing autoimmune myopathies; SRP, signal recognition particle. [file ar4468-S3.pdf]

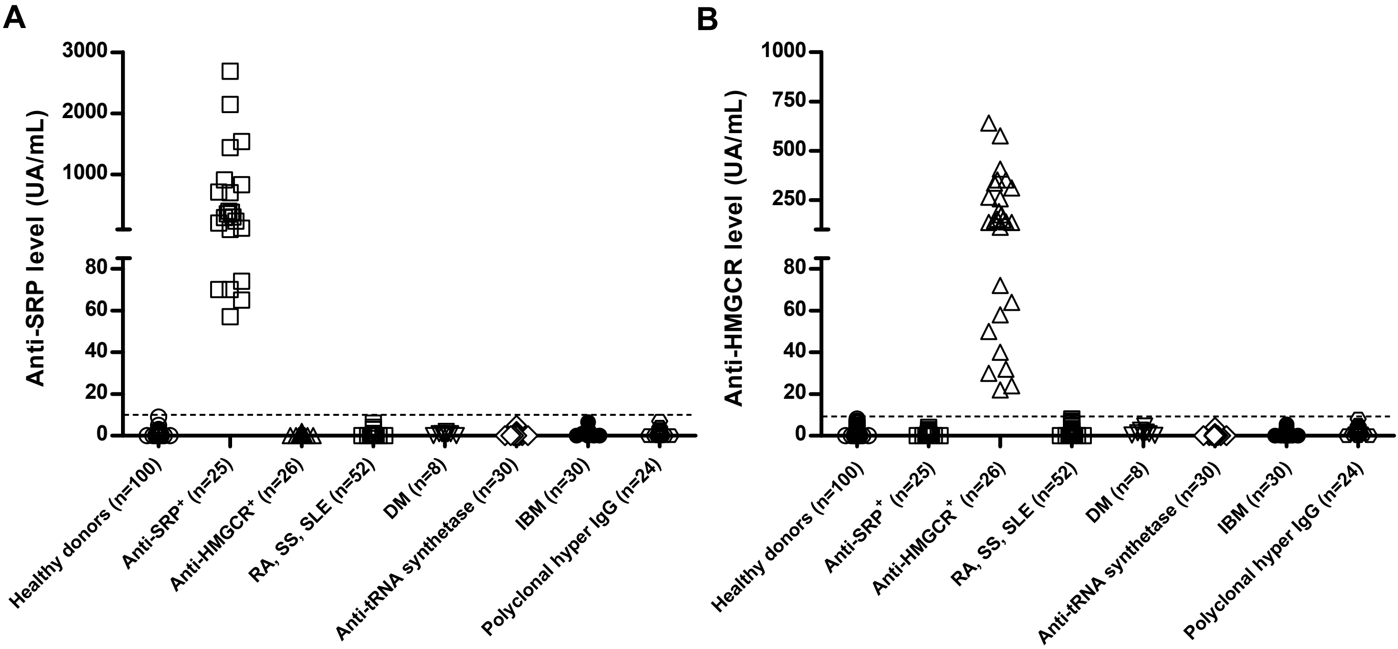

Additional file 3 Validation of ALBIA-NAM. A positivity cutoff of the multiplex assay (dotted line) was determined as the 99th percentile of the healthy donors distribution (open circles) for both **(A)** anti-SRP (9 AU/mL) and **(B)** anti-HMGCR aAbs (8 AU/mL). Sera from patients with different inflammatory/autoimmune conditions including rheumatoid arthritis (RA), systemic sclerosis (SS), systemic lupus erythematosus (SLE), dermatomyositis (DM), anti-tRNA synthetase aAb positive myositis or inclusion body myositis (IBM), as well as patients with polyclonal hyper-gammaglobulinemia were assayed.
